# Implications of methodological differences in measuring the rates of exclusive breastfeeding in Nepal: findings from literature review and cohort study

**DOI:** 10.1186/s12884-016-1180-9

**Published:** 2016-12-12

**Authors:** Vishnu Khanal, Andy H. Lee, Jane A. Scott, Rajendra Karkee, Colin W. Binns

**Affiliations:** 1 Nepal Development Society, Bharatpur, Nepal; 2 School of Public Health, Curtin University, Perth, Australia; 3 School of Public Health and Community Medicine, BP Koirala Institute of Health Sciences, Dharan, Nepal

**Keywords:** Cohort study, Exclusive breastfeeding rate, Infant feeding, Nepal, Review

## Abstract

**Background:**

Correct measurement and continuous monitoring of exclusive breastfeeding are essential to promote exclusive breastfeeding. Measuring exclusive breastfeeding is a complex issue as rates can vary according to the definition, measurement period, questions asked, and infant’s age. This article reviewed the methodology of reporting exclusive breastfeeding in Nepal, and compared exclusive breastfeeding rates using data from a cohort study undertaken in western Nepal.

**Methods:**

A literature review was first conducted on studies published during 2000–2014. In our cohort study, 735 mother-infant pairs were recruited within the first month postpartum and followed up during the fourth and sixth months.

**Results:**

The majority of studies in Nepal, including national surveys, used the World Health Organization (WHO) recommended definition (only breastmilk with the exception of medicine and vitamin syrup), and the most common measurement period was a 24-h recall. Our data demonstrated that the exclusive breastfeeding rate during the sixth month was 8.9% using the recall-since-birth method but was 18.7% using the 24-h recall method. Substantial differences in rates were also found during the first (66.3% vs 83.9%) and fourth months (39.2% vs 61.1%).

**Conclusion:**

We found that recent studies reporting exclusive breastfeeding in Nepal varied considerably in methodology. The most commonly used measurement, the 24-h recall, leads to over-estimation of the prevalence of exclusive breastfeeding when compared to the recall-since-birth method. A common standard of reporting exclusive breastfeeding is clearly needed for evidence-based decision making.

**Electronic supplementary material:**

The online version of this article (doi:10.1186/s12884-016-1180-9) contains supplementary material, which is available to authorized users.

## Background

Exclusive breastfeeding offers many short and long-term health and nutrition benefits. In the short term, it is the best source of nutrition, and supports optimum growth and development of the infant [1]. In the long term, exclusive breastfeeding is likely to protect from obesity, type-2 diabetes, and is associated with increased intelligence quotient scores [2, 3]. Exclusive breastfeeding impacts on the infant gut microbiome which in turn may contribute to the programming of infant metabolism and immune function [4]. In low and middle income countries where supply of clean water is limited and hygiene of the mother and child is poor, substituting breastmilk with other fluids or food is likely to introduce pathogens resulting in infection-related infant mortality and morbidity [5]. Furthermore, the introduction of other fluids and foods reduces the frequency of breastfeeding and contributes to reduced milk production, ultimately affecting milk supply [6]. A recent systematic review showed that there were higher rates of infant deaths among 0-5 months infants who were not breastfed, partially breastfed or predominantly breastfed compared to those who were exclusively breastfed [7]. Similarly, Victora et al.[8] projected that the scaling up of optimal breastfeeding practices could save 823000 deaths annually among children under five years of age.

The World Health Organization's (WHO) *Comprehensive implementation plan on maternal, infant and young child nutrition* was endorsed in 2012 and identified six targets to reduce nutrition-related mortality and mortality [9]. Of these, target 5 was by 2025, to increase the rate of exclusive breastfeeding to 50% in the first six months from 37% for the period 2006–2010 [9]. ‘This would involve a 2.3% relative increase per year and would lead to approximately 10 million more children being exclusively breastfed until six months of age’ ([9], p. 9). To measure the progress of member states in achieving this target it is essential to monitor the practice of exclusive breastfeeding regularly and consistently.

Measuring exclusive breastfeeding however, is a complex issue because the rate can vary with respect to the recall duration, questions asked, age of infant and definition adopted [10–12]. WHO originally defined exclusive breastfeeding as ‘infant has received only breastmilk from his/her mother or wet nurse, or expressed breastmilk, and no other liquids or solids with the exception of drops or syrups consisting of vitamins, mineral supplements or medicine’ [13]. In 2007, this definition was modified to allow a child to receive oral rehydration salts [14]. However, this strict definition of exclusive breastfeeding often is not applied in studies which purport to report levels of exclusive breastfeeding [10, 12, 15], making it difficult to compare the findings of studies both within and between countries.

One of the major issues with measuring exclusive breastfeeding is accounting for prelacteal feeding, which is common practice in countries in South Asia, [16, 17] where babies may receive prelacteal feeds for the first few days of life but after which mothers typically revert to exclusive breastfeeding for several months at least. However, accounting for prelacteal feeding is essential for some purposes due to possible infections in early infancy and the loss of the gut priming effect of colostrum as the first feed [4]. Nevertheless, including prelacteal feeding would dramatically reduce the exclusive breastfeeding rate since birth and suggest that a large proportion of infants had never been exclusively breastfed [12].

The prevalence of exclusive breastfeeding also can vary widely depending on the indicator measure used. The indicator favoured by the WHO employs the 24-h recall methodology to determine the proportion of infants 0–5 months of age who received only breastmilk during the previous day [14]. This method has been adopted in countries where capacity and resources are limited and used in household surveys such as the Demographic and Health Surveys [18]. The 24-h recall method can lead to over-estimation due to its inability to capture prelacteal feeding and intermittent use of complementary feeds. That is, infants who only received breastmilk the previous day may have received other foods before that time [19]. On the other hand, measurements based on recall-since-birth can be affected by recall error. For instance, while maternal recall of initiation and duration of breastfeeding is generally valid and reliable over a short period (≤3 years), recall of the age of introduction of other foods and fluids is less reliable [20]. Therefore, duration of exclusive breastfeeding is best measured prospectively using cohort methodology with short recall intervals [21, 22]. Binns et al. [22] reported wide discrepancies in the rates of exclusive breastfeeding in China and Japan when the results of national and regional cross-sectional surveys which predominantly used the 24-h recall method were compared with data prospectively collected in separate studies using the recall-since-birth method. However, comparison of exclusive breastfeeding rates between the two methods using the same data source is rarely made. Significant differences between the two methods of more than 40 percentage points at two and four months of age were first observed in a study conducted in Sweden [19]. To our knowledge however, there has only been one report of a comparison study from a South Asian country reported in 2009 [23].

One of the strategies to reduce under nutrition in Nepal is promotion of exclusive breastfeeding for the first 6 months [24]. Monitoring of the success of this strategy requires surveillance data on exclusive breastfeeding ‘for six months’. However, the current knowledge of exclusive breastfeeding in Nepal is based almost entirely on cross-sectional studies [25–27] which likely overestimate the true rate of exclusive breastfeeding. The only study that has reported exclusive breastfeeding using prospectively collected data were from the Kaski district of Nepal [28]. To date, there has been no study that specifically addressed issues such as definition of exclusivity, duration of measurement, and age composition of infants, all of which can contribute to the reported rate variations in Nepal and the South Asian region. Further research to critically analyse the existing breastfeeding studies is deemed necessary for accurate monitoring and appropriate reporting of such data in the future. Such critical appraisal would also inform future breastfeeding research in Nepal and other countries in South Asia. The objectives of this study are: (1) to review the definitions and methods of reporting exclusive breastfeeding in Nepal used during the period 2000–2014; and (2) assess the magnitude of differences in exclusive breastfeeding rates between the 24-h recall and recall-since-birth methods using a single data set from a prospective cohort study undertaken recently in western Nepal.

## Methods

**Fig. 1:**
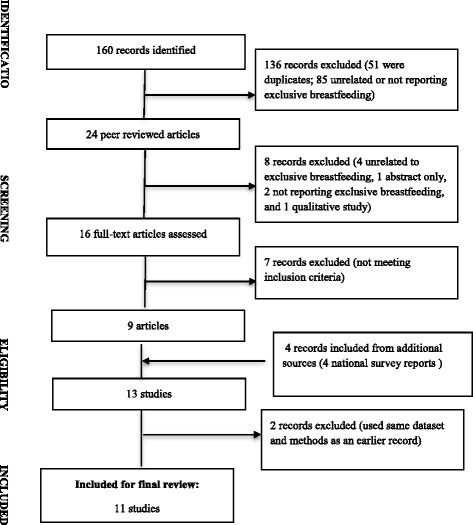
PRISMA flow chart for review of exclusive breastfeeding studies in Nepal, 2000–2014

Our paper presents findings from two different methods; a literature review and a large community-based prospective cohort study. Firstly, we conducted a literature review using the Preferred Reporting Items for Systematic Reviews and Meta-Analyses (PRISMA) Guidelines [29]. A comprehensive literature search was conducted in PubMed, the Cumulative Index to Nursing and Allied Health Literature, and Web of Science databases, with key words ‘breastfeeding’, ‘breast-feeding’ or ‘breast feeding’ or ‘infant feeding’, and ‘Nepal’. Inclusion criteria were: (1) articles published during 2000–2014; (2) reporting exclusive breastfeeding; (3) study conducted in Nepal; and (4) published in the English language. We excluded those articles reporting qualitative findings, but included data sources which are the major policy informing tools in Nepal, namely: (1) Nepal Demographic and Health Surveys (NDHS 2001, 2006, and 2011); and (2) Multiple Indicator Cluster Survey (MICS) [30]. In addition, we performed a manual reference search of identified articles. Figure 1 describes the process of selecting the articles for the review. Two terms that explain the literature review summary are ‘indicator measure’ and ‘age of the infant’. We defined indicator measure as the methods which were used to measure exclusive breastfeeding such as 24-h recall and recall-since-birth. Age of the infant refers to the infant’s age at which data were collected. This was important as younger infants (e.g. < 3 months) are more likely to be exclusively breastfed than their older counterparts. Such variation in infant’s age affects the reported breastfeeding rates.

**Fig. 2:**
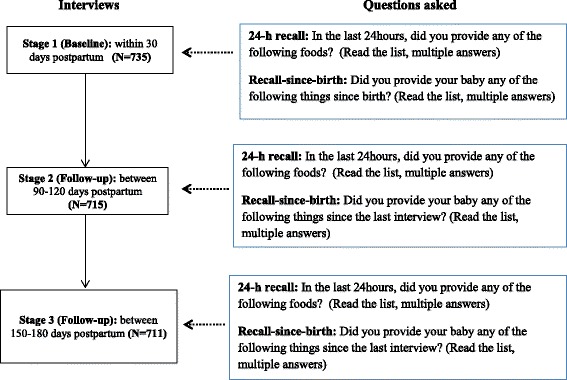
Study interview flow chart and questions asked to measure breastfeeding rates

Secondly, we used data from a prospective cohort study of infant feeding practices conducted during January-October 2014 in the Rupandehi district of western Nepal. This district is located in the south-western plain areas (Terai) of Nepal bordering India in the south. The methodology of this study has been described previously [31, 32] but briefly 735 mother-infant pairs (rural 378, urban 357) were randomly recruited from 15 rural and 12 urban locations. Mothers were recruited from their communities within 30 days of giving birth and followed up during the fourth (90–120 days) and sixth months (150–180 days) (Fig. 2). The main outcome, exclusive breastfeeding, allowed breastmilk, vitamin or mineral syrups, medicine and oral rehydration salt, but no other liquid or solid/semi-solid food, in accordance with the current WHO definition [14]. We used two methods (24-h recall and recall-since-birth) to estimate the rate of exclusive breastfeeding during the first, fourth, and six months after delivery. For the recall-since-birth method, information was obtained based on recall-since-birth at the first interview, and recall-since-the last interview during the fourth month and sixth month interviews. Information was collected through a questionnaire adapted from the NDHS [25] and an earlier cohort study [28] which were in accordance with the WHO’s *Indicators for assessing infant and young child feeding practices part 2: measurement* [33]. Pre-testing was done and only a few minor changes were needed to make our questionnaire understandable in our study setting. A list of common food items was read to mothers to help them remember their infant feeding practices. The questionnaire to collect this information is provided as a web appendix (Additional file 1). Data were collected by female enumerators who had vocational training in health science after high school level of education. These enumerators received a one-day orientation on data collection including a data collection exercise in the community and the researcher provided feedback after the pretesting. The enumerators were regularly monitored by the first author to ensure the quality of data. During the follow-up interviews, enumerators again received a brief orientation as most of the questions on infant feeding were repeated. The interviews were conducted in the Nepali language.

We defined exclusive breastfeeding within the last 24-h as: mother did not introduce any food item besides her breastmilk or other women’s milk [33].

A mother was classified as exclusive breastfeeding during the first month using recall-since-birth if (1) she did not introduce any prelacteal feeds (2) responded that she had been feeding only breastmilk since birth and (3) was exclusively breastfeeding based on 24-h recall. A mothers was classified as exclusively breastfeeding during the fourth month using recall-since-birth if (1) she had exclusively breastfed during the first month, (2) did not introduce any complementary food since the last interview (confirmed reading list) and (3) did not introduce any complementary food in the last 24-h (confirmed reading list). Similarly, a mothers would be classified as exclusive breastfeeding during the sixth month based on recall-since-birth if (1) she had been exclusively breastfeeding during the fourth month, (2) did not introduce any complementary food since last interview (confirmed reading list) and (3) did not introduce any complementary food in the last 24-h (confirmed reading list). Any mother who was not breastfeeding at the time of interview was classified as not exclusively breastfeeding.

Ethics approval was obtained from the Human Research Ethics Committee of Curtin University (HR 184/2013) and the Nepal Health Research Council. Mothers provided written consent for themselves and their infants, and personal identifiers were removed before analysis. When mother could not read and write, enumerators read the consent form to them. Mothers provided a thumb print as their initials if they agreed to participate. This method was approved by both ethics committees.

## Results

### Selection of studies in the review

Our literature review found only 9 articles which reported exclusive breastfeeding in Nepal. In addition, three NDHS, and one MICS were subsequently included. On reviewing the articles in full, it was found that two studies [34, 35] had analysed the same dataset and used the same methods (e.g. indicator measure and definitions) as in the NDHS 2006 [27] therefore, these papers were removed from the review leaving 11 studies (Fig. 1). Of these 11 studies, 10 used unique datasets and one used the NDHS 2006 and 2011 but analysed the data differently from the original NDHS 2006 report using the WHO 2007 definition of exclusive breastfeeding. Only one of them was a cohort study [28] and the rest were cross-sectional studies.

### Definitions used in measuring exclusive breastfeeding

Four national surveys (NDHS and MICS) [25–27, 30], one secondary analysis of NDHS 2006 and 2011 [36], and two other studies [37, 38] followed the WHO definition of exclusive breastfeeding of the time. It should be noted that the 2001 and 2011 NDHS defined exclusivity as “only breastmilk”. The 2006 NDHS did not provide the actual definition in the report, but advice from researchers involved confirmed that they followed the WHO 1991 definition [13] (personal communication, Mr. Sujan Karki, March, 2015). Two studies [28, 39] used a slight variant of the WHO definition and described exclusive breastfeeding as ‘nothing else (except medicines) but breast milk’ and ‘given breastmilk only without any other feeds (aside from medications) since birth’, respectively. Two cross-sectional studies [40, 41] did not give any definition.

### Indicator measure

There were variations in the indicator measure or methods of reporting exclusive breastfeeding used to estimate prevalence of exclusive breastfeeding. Six studies used the 24-h recall measure [25–27, 30, 36, 41]. One study used recall-since-birth using repeated measures in a prospective cohort study up to 6 months [28], and one used recall-since-birth at two months [39]. Two studies used retrospective recall of mothers of infants aged 6 to 23 months [37] and 9 months postpartum [38], and asked mothers how long they had breastfed exclusively.

### Age of infant

Age of infant is another important aspect as younger infants are more likely to be breastfed exclusively than their older counterparts. Whilst most studies reported on infants aged 0–5 months [25–27, 30, 36, 41], one study [40] estimated exclusive breastfeeding rate at the 5^th^ month. On the other hand, the study conducted by Subedi et al. [37] reported on the prevalence of breastfeeding up to six months among infants aged 6 to 23 month, while Chandrashekar et al. [39] considered young infants less than 2 months, and Karkee et al. [28] reported exclusive breastfeeding rates at 1, 4, and 6 months.

### Reported exclusive breastfeeding rates in Nepal

In view of the above methodological discrepancies, we found large variations in the rates of exclusive breastfeeding between studies. With the exception of one prospective cohort study which observed a low rate (29.7%) of exclusive breastfeeding at 6 months (22 weeks) using recall-since-birth [28], other studies reported a higher prevalence of greater than 50% amongst infants less than 6 months [37, 39, 40]. Ullak et al. [38] reported 9% exclusive breastfeeding prevalence at 6 months.

### Comparison of exclusive breastfeeding rates in a cohort study using 24-h recall and recall-since-birth methods

We used data from our cohort study to compare exclusive breastfeeding rates during the first, fourth, and sixth months (Fig. 2); results are presented in Table 1. All infants were breastfed at the time of recruitment. Almost one-third of infants were provided with prelacteal feeds, leaving only 69.4% of them being exclusively breastfed at their first feed. The rates of exclusive breastfeeding are substantially different between the 24-h recall and the recall-since-birth methods at all three time points. During the first month, the prevalence of exclusive breastfeeding based on the 24-h recall method was 83.9% but was 66.3% using recall-since-birth, which accounted for prelacteal feeds. Similarly, the exclusive breastfeeding rate during the sixth month was 18.7% from the 24-h recall method and was half of that (8.9%) using recall-since-birth.

**Table 1:** Prevalence of exclusive breastfeeding from a cohort study using two measurement indicators, Nepal, 2014

Exclusive breastfeeding	1^st^ month		4^th^ month		6^th^ month	
Method	24-h recall (*N* = 735)	recall-since-birth (*N* = 735)	24-h recall (*N* = 715)	recall-since-birth (*N* = 715)	24-h recall (*N* = 711)	recall-since-birth (*N* = 711)
Rate (%)	617 (83.9%)	487 (66.3%)	437 (61.1%)	280 (39.2%)	133 (18.7%)	63 (8.9%)
95% confidence interval	81.3% to 86.6%	62.8% to 69.7%	57.5% to 64.7%	35.6% to 42.7%	15.8% to 21.6%	6.8% to 11.1%

## Discussion

This study reviewed and compared the definition and methods of reporting exclusive breastfeeding in Nepal. We found three main issues related to the measurement and reporting of exclusive breastfeeding namely, inconsistent definition of exclusive breastfeeding, indicator measurement and interpretation of the WHO indicator based on 24-h recall.

Data collected from the same participants in our cohort study were used to demonstrate the differences in exclusive breastfeeding rates between methods. Our findings suggest that the extensive use of NDHS data based on 24-h recall would inevitably over-estimate the prevalence of lifelong exclusive breastfeeding. In other words, estimates of exclusive breastfeeding rates in Nepal are probably much lower than those previously reported. Such differences in rates between recall-since-birth and 24-h recall have also been demonstrated by others [19, 22, 23]. As in any observational study which relies on self-reported data there is a potential in our study for recall bias and hence the actual duration of exclusive breastfeeding may differ from that reported. However, our data were collected prospectively with relatively short recall intervals, thereby reducing the likelihood of recall bias.

In surveys such as NDHS and MICS, age group of infants is another concern when 0–5 month old infants are aggregated to be the denominator for the 24-h recall prevalence. In our cohort study, exclusive breastfeeding during the sixth month was 18.7% by 24-h recall. For illustration purposes, let us regard the study as a cross-sectional survey that had similar number of infants in three age groups: (1 (*n* = 735), 4 (*n* = 715), and 6 (*n* = 711) months), i.e. 2,161 infants in total available for interview from Table 1. The number of infants being exclusively breastfed based on 24-h recall in each age group would be 617 + 437 + 133 = 1,187, giving a prevalence of 54.9% which is comparable to those of previous studies in Table 2. This is not however the proportion of infants exclusively breastfed for the entire six month period, which is how cross-sectional data derived by this method often are misinterpreted [11, 12, 23]. This difference has been demonstrated also in two other cross-sectional studies conducted in Nepal and East-Timor [36, 42] where the actual 24-h prevalences of exclusive breastfeeding at the sixth month (33.1% and 24.9%, respectively) was lower than the commonly reported 0–5 month exclusive breastfeeding prevalence of 66.3% and 49%, respectively. If infants should be breastfed for six months according to the WHO recommendation, then the indicator must reflect ‘exclusive breastfeeding for six months’.

**Table 2:** Studies reporting exclusive breastfeeding in Nepal, 2000–2014

Reference	Study design	Source of data and sample size	Definition of exclusive breastfeeding	Indicator measure	Reported EBF rate
Peer reviewed publications					
Chandrashekhar et al. [39]	cross-sectional	primary data (*n* = 385)	nothing else (except medicines) but breast milk	recall-since-birth	82.3% among <2 months infants
Subba et al. [40]	cross-sectional	primary data (*n* = 168)	not detailed	not reported	60.5% at 5^th^ month
Subedi et al. [37]	cross-sectional	primary data (*n* = 261)	WHO, 2007 definition	recall at 6–23 month	81.6% upto 6 months
Ulak et al. [38]	cross-sectional	primary data (*n* = 325)	WHO, 2007 definition	recall-at-9^th^ month	at 1^st^, 3^rd^ and 6^th^ months were 74%, 24% and 9%
Khanal et al. [16]	cross-sectional	NDHS 2006 (*n* = 482) and NDHS 2011 (*n* = 227)	WHO, 2007 definition	24-h recall	53.2% in 2006 and 66.3% in 2011 among 0–5 month infants
Locks et al. [41]	cross-sectional (subset of randomized control trial)	primary data (*n* = 750)	not reported	24-h recall	65% among 0–5 month infants
Karkee et al. [28]	cohort	primary data (*n* = 639)	given breastmilk only without any other feeds (aside from medications) since birth	recall-since-birth	84.4% during first month, 67.2% during 3 months, and 29.7% EBF at 6^th^ month
National surveys					
Ministry of Health and Population et al. (2001) (NDHS 2001) [26]	cross-sectional	primary data (*n* = 648)	WHO, 1991 definition	24-h recall	68.3% among 0–5 month infants
Ministry of Health and Population et al. [27] (NDHS 2006)	cross-sectional	primary data (*n* = 477)	WHO,1991^a^	24-h recall	53.0% among 0–5 month infants
Ministry of Health and Population et al. [25] (NDHS 2011)	cross-sectional	primary data (n = 537)	WHO, 1991	24-h recall	69.6% among 0–5 month infants
Central Bureau of Statistics et al. (2010) (MICS) [30]	cross-sectional	primary data (*n* = 452)	WHO, 2007 definition^a^	24-h recall	63.9% among 0–5 month infants

*EBF* exclusive breastfeeding, *NDHS* Nepal Demographic and Health Survey, *MICS* Multiple Indicator Cluster Survey

^a^from personal communication with researcher involved in NDHS and MICS 4

Traditionally the use of prelacteal feeds has been a common practice in Nepal, and more recently the use of formula feeding is becoming increasingly prevalent [43]. Previous studies have reported that the introduction of prelacteal feedings or early supplementation of food or fluid is likely to interfere with normal gut microbiome [4], introduce infections [44] and interfere with the duration of breastfeeding. While, it is sometimes claimed that accounting for prelacteal and a few intermittent feeds can greatly under-estimate the rate of exclusive breastfeeding [12], ignoring prelacteal feeding would lead to losing focus on the harmful effects of prelacteal feeds as well as the early supplementation of infant formula. For instance, in our study, if prelacteal feeds were ignored, the rate of exclusive breastfeeding at first feed would be 100% as there was universal breastfeeding initiation [31]. Such interpretation of breastfeeding is also likely to mislead breastfeeding promotion programs providing a false sense of security.

Furthermore, although most studies in the past claimed to adhere to the WHO definition of exclusive breastfeeding [13, 14], we found they did not follow the definition exactly, and particularly with respect to measurement using the 24-h recall and recall-since-birth methods. Consequently, it is difficult to compare rates across studies. Similar comparability problems were also noted in Japan [45] and Australia [15] .

For health policy advocacy and planning, consistency in definition of exclusive breastfeeding and the measurement indicator is necessary in order to monitor and compare the changes in exclusive breastfeeding rates over time, across regions and between population subgroups. In addition, future research in breastfeeding needs to provide the evidence that aligns with the target of the Ministry of Health Nepal [24] and the 2025 Global Targets [9]. As a priority, future research should report both the point-in-time (24-h recall method) and life-long data (recall-since-birth method) [12] to estimate the 24-h prevalence and rate of exclusive breastfeeding for 6 months, respectively and should be used in conjunction with a well-designed cohort study taking repeated measurements. While this method is known to be more resource intensive, some sentinel sites could be established to represent the country, and prospective data could be collected periodically from these chosen sites to measure the rates of exclusive breastfeeding in Nepal [22].

The 24-h recall method is commonly adopted because of its feasibility in large nationally representative studies for evidence-based decision making in developing countries including Nepal [18, 25, 46] and is likely to continue in nationwide Demographic and Health Surveys. However, it should be noted that the statistics generated from these studies do not provide reliable data for monitoring exclusive breastfeeding rates for six months. Therefore, the resulting estimate should be reported as the ‘24-h prevalence of exclusive breastfeeding’ to avoid misinterpretation. It is recommended to increase the sample size in each infant age group from newborn up to six months. This will enable accurate reporting of the 24-h prevalence as well as the proportion of infants being exclusively breastfed according to infant age with a high power to detect any apparent age-related changes and patterns.

## Conclusion

This study found that the use of the 24-h-recall measurement indicator significantly over-estimates life-long exclusive breastfeeding rates. To facilitate uniform and accurate reporting of exclusive breastfeeding rates and monitor national targets for breastfeeding, future efforts should be on reporting exclusive breastfeeding based on the recall-since-birth method, using a cohort study design and repeated measurement to collect infant feeding information.

## Additional file

Additional file 1: Web Appendix: Questions asked collect breastfeeding information. (DOCX 23 kb)

## Abbreviation

MICS: Multiple Indicator Cluster Survey
NDHS: Nepal Demographic and Health Surveys
PRISMA: Preferred Reporting Items for Systematic Reviews and Meta-Analyses
WHO: World Health Organization
