# Supplementary material for: Implications of methodological differences in measuring the rates of exclusive breastfeeding in Nepal: findings from literature review and cohort study
Source: BMC Pregnancy Childbirth. 2016 Dec 12;16:389. doi: 10.1186/s12884-016-1180-9 (PMC5154002; doi:10.1186/s12884-016-1180-9)
Supplement: Additional file 1: — Web Appendix: Questions asked collect breastfeeding information. (DOCX 23 kb) [file 12884_2016_1180_MOESM1_ESM.docx]

**Baseline Questionnaire**

**Participant’s identification and basic information**

| Respondent ID /______/_______/_______/  Village/Ward ID /______/_______/  Interviewer ID /_______/_______/  Date of interview Month…………Day……………  Contact phone: …….  Next to kin: ………………..  Are of residence: (i) Rural (ii) Urban | Name of the mother …………………………………………….  Sex of child:  Name of child:  Age of the infant .............days  Height of the mother (in Centimetres)……………………  Mode of delivery: Vaginal / Instrumental/ Caesarean |
| --- | --- |

*If the age of the infant is more than 30 days, stop this interview.*

**Breastfeeding**

| 116 | Have you ever breastfed NAME (child)? | O: No  1: Yes |  |
| --- | --- | --- | --- |
| 117  A  B | Are you currently breastfeeding?  If Yes, how long are you intending to breastfeed?  If No, how long did you breastfeed? | O: No  1: Yes  …………..months……… year/s  ………………..days |  |
| 118 | How long after birth did you first breastfeed NAME (the child)? | 1. Less than or within 1 hour 2. After1 hr till 6 hours 3. 7-24 hours 4. 1-3 days. 5. 3 days or more 6. Not yet 7. Do not know |  |
| 119 | Before you initiated to breastfeed, did you provide NAME (child) anything aside from breast milk?  *( Please read the list to help mother to recall)*  **Decide if mother provided prelacteal feeds** | 1. Only mother’s milk is provided 2. Plain water 3. Cows’ or buffalos’ milk 4. Sugar/glucose water 5. Sugar/salt water 6. Ghee 7. Honey 8. Ghee and honey 9. Fruit juice 10. Infant Formula 11. Breast milk from other mothers 12. Other (specify)……………. 13. Not provided 14. Provided |  |
| 120  A  B  C | If you have given any food to NAME (of baby) other than breast milk for the first time, why did you do so?  What do you think is the benefit of providing the foods other than breast milk **(. …………pick the name from list of Q 119)** as the baby’s first food?  In your opinion, is there any harm in providing such foods **(…… *pick up the name Q119*)** from the list above?  *( more than one answer possible)*  *(Do not read the options )*  Who advised you to provide such foods **(..…..*pick the name of food from above listQ119* )** to your baby?  *( more than one answer possible)*  *(Do not read the options )* | 1. Was not able to breastfeed after childbirth due to exhaustion 2. Was not able to breastfeed as I has caesarean delivery 3. This is our culture that we first provide other foods to our babies 4. Other reason……..   …………………………………………………………………………………………………………………………………………………………………..…………………………………   1. I do not think there any harm 2. Increased chances of infection 3. Increased diarrhoea 4. Difficult to establish breastfeeding after providing other foods 5. Others ………………………………. 6. My mother/Mother-in-law 7. Nurse/ ANM 8. Female Community Health Volunteers 9. Traditional attendant 10. Senior women 11. Doctor/Health Assistant/CMA 12. Others ……….. 13. No one |  |
| 121  A  B | Did you provide the yellowish first breastmilk *(Nepali Term: Bigauti dudh)* to NAME (your baby)?  ***If No: ask the following questions***  What was the reason for not giving colostrum for the child?  Who told you to not to provide colostrum NAME? | 1.Yes  0: No   1. Colostrum is not digestible for the infant 2. Colostrum is harmful for the infant 3. Colostrum is not clean enough to provide the infant 4. Colostrum does not look nice (Pus like appearance) 5. Others…………………………… 6. My mother/mother-in-law 7. Nurse/midwife/MCHW 8. FCHV 9. Traditional attendant/Dai 10. Senior women 11. Doctor/ HA/CMA 12. Others……………… 13. No one |  |
| 122 | Did you provide your baby any of the following food or drink **since birth?**  ***(Read the list , multiple answers)*** | 1. Nothing other than mother’s breastmilk 2. Infant formula (Cerelac/Nestle) 3. Plain water 4. Cow or buffalo’s milk 5. Breast milk from another woman 6. Sugar water 7. Sugar salt water 8. Ghee 9. Honey 10. Tea 11. Adult foods 12. Other food perceived good for child (write the name if any) …………………. |  |
| 123 | **In last 24hours,** did you provide any of the following foods?  (Read the list, multiple answers) | 1. Nothing other than mother’s breastmilk 2. Infant formula (Cerelac/Nestle) 3. Plain water 4. Cow or buffalo’s milk 5. Breast milk from another woman 6. Sugar water 7. Sugar salt water 8. Ghee 9. Honey 10. Tea 11. Adult foods 12. Other food perceived good for child (write the name if any) ………………….   …………………. | If option 1 and/or 5 (Only)- go to **Q 130** ….. |
| 124 | How old was NAME (child) when you introduced the food (other than breast milk)? | …………weeks |  |

**Follow up questionnaire: during fourth month**

**Participant’s identification and basic information**

| Interviewers ID /______/_______/_______/  Village/Ward ID /______/_______/  Date of Interview: ……………….  Place of residence: 1 Rural 2 Urban | Name of the mother ……………………………………  Age of the child .............Months  Sex of child:…………  Name of child:……………  Date of birth of child: ……………. |
| --- | --- |

| 201 | Are you currently breastfeeding? | No  Yes- ………….**Go to Q 204** |  |
| --- | --- | --- | --- |
| 202 | If No, how long did you breastfeed? | ……weeks …….months. |  |
| 203 | If discontinued breastfeeding:  Why did you discontinue breastfeeding?  ***(More than one answer possible)*** | 1. Baby was crying 2. Baby did not gain weight 3. My breastmilk was insufficient 4. I had to go to work 5. I was tired and fatigued 6. I did feel well (illness) 7. My family members advised me to do so 8. Other reasons ………………. | **Go to Q 208** |
| 204 | Did you provide your baby any of the following things since the last interview?  (read the list) | 1. Nothing provided except my (or someone else’s) breastmilk 2. Vitamin syrup/ ORS (Jeevanjal)/Medicine (for illnesses) 3. Plain water 4. Cows’ or buffalos’ milk 5. Porridge 6. Superflour porridge or mixture of cereal and rice. 7. Sugar water 8. Sugar salt water 9. Ghee 10. Honey 11. Ghee and honey 12. Tea 13. Adult foods 14. Infant formula such as cerelac 15. Other food perceived good for child (write the name if any) …………………………… | If 1 and or 2…..  **Go to Q207** |
| 205 | IF you have provided any thing other than breastmilk and medicine, when did you start to do so | Infant’s age:………weeks …….... months |  |
| 206 | If you provided other foods other than breastfeeding:  Why did you introduce other foods?  ***(More than one answer possible)*** | 1. Baby was crying 2. Baby did not gain weight 3. My breast milk was insufficient 4. I had to go to work 5. I was tired and fatigued 6. I didnot feel well (illness) 7. My family members advised me to do so 8. Other reasons …………………… |  |
| 207 | In the last 24 hours, did you give any of following food to your baby (NAME) | 1. Nothing provided except my (or someone else’s) breastmilk 2. Vitamin syrup/ ORS (Jeevanjal)/Medicine (for illnesses) 3. Plain water 4. Cows’ or buffalos’ milk 5. Porridge 6. Superflour porridge or mixture of cereal and rice. 7. Sugar water 8. Sugar salt water 9. Ghee 10. Honey 11. Ghee and honey 12. Tea 13. Adult foods 14. Infant formula such as cerelac 15. Other food perceived good for child (write the name if any) | **If other than 1 or 2: please go back to Q205 and 206** |

**Follow-up during sixth month**

**Participant’s basic information**

| Respondent ID /______/_______/_______/  Village/Ward ID /______/_______/  Date of Interview Month…………………Day…………  Phone number of respondent: ……………. | Name of the mother …………………………………………….  Sex of child:  Age of the child .............months  Name of child: ………………..  Date of birth of child: ……………. |
| --- | --- |

| 301 | Are you currently breastfeeding? | 1. No 2. Yes- ………**Go to …..Q304** |  |
| --- | --- | --- | --- |
| 302 | If no, how long did you breastfeeding? | …………….weeks…..months |  |
| 303 | If discontinued breastfeeding:  Why did you discontinue breastfeeding?  **(More than one answer possible)** | 1. Baby was crying 2. Baby did not gain weight 3. My breast milk was insufficient 4. I had to go to work 5. I was tired and fatigued 6. I didnot feel well (illness) 7. My family members advised me to do so 8. Other reasons…..   **…….Go to Q 308** |  |
| 304 | If you are currently breastfeeding,  Did you provide your baby with any of the following things **since the last interview?** | 1. Nothing provided except my (or someone else’s) breastmilk 2. Vitamin syrup/ ORS (Jeevanjal)/Medicine (for illnesses) 3. Plain water 4. Cows’ or buffalos’ milk 5. Porridge 6. Super flour porridge or mixture of cereal and rice. 7. Sugar water 8. Sugar salt water 9. Ghee 10. Honey 11. Ghee and honey 12. Tea 13. Adult foods 14. Infant formula such as cerelac 15. Other food perceived good for ……… | **If response 1and 2: Go**  **to Q 307** |
| 305 | *If provided food other than mother’s milk,*  When did you introduce? | Child’s age…weeks…..months |  |
| 306 | Why did you introduce food other than breastmilk? | 1. Baby was crying 2. Baby did not gain weight 3. My breast milk was insufficient 4. I had to go to work 5. I was tired and fatigued 6. I didnot feel well (illness) 7. My family members advised me to do so 8. Other reasons…. |  |
| 307 | **In last 24hours,** did you provide any foods beside breast milk?  *Interviewer: read the list above and confirm none of the food listed above were given by probing.* | 1. Nothing provided except my (or someone else’s) breastmilk 2. Vitamin syrup/ ORS (Jeevanjal)/Medicine (for illnesses) 3. Plain water 4. Cows’ or buffalos’ milk 5. Porridge 6. Super flour porridge or mixture of cereal and rice. 7. Sugar water 8. Sugar salt water 9. Ghee 10. Honey 11. Ghee and honey 12. Tea 13. Adult foods 14. Infant formula such as cerelac 15. Other food perceived good for ………   If provided other than no.1 and 2………..**Go to back and complete Q 305 and 306** |  |

**Note:** *The questions available in this web appendix are only related to figure 2 and methodology of this manuscript. Full list of questions asked during the project “Breastfeeding practices and lactation mastitis in Western Nepal: A prospective cohort study” will be made available through Institutional Repository of Curtin University Library (Open access).*
